# Supplementary material for: Isoenzymes of the Flavonoid and Phenylpropanoid Pathways Show Organ-Specific Regulation during Apple Fruit Development
Source: Int J Mol Sci. 2023 Sep 20;24(18):14353. doi: 10.3390/ijms241814353 (PMC10532258; doi:10.3390/ijms241814353)
Supplement: Supplementary file 1 [file ijms-24-14353-s001.zip › Figure S4.pdf]

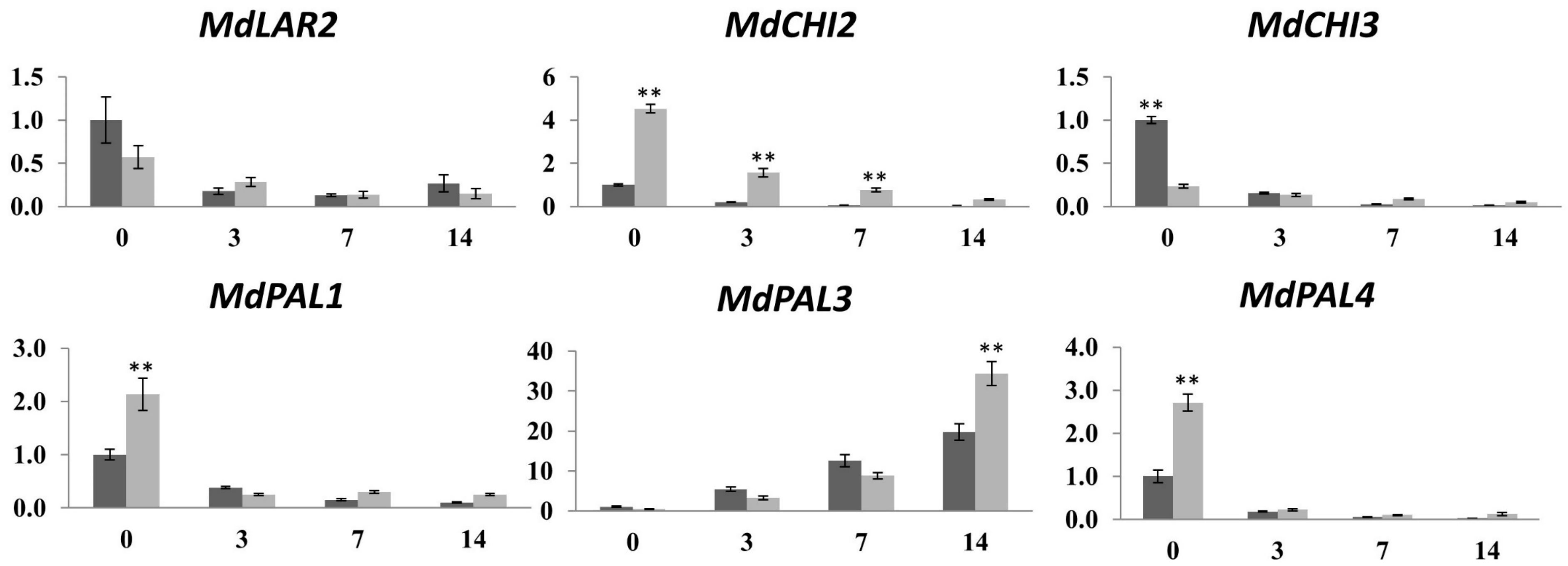

Figure S4. Relative expression levels of apple genes showing similar trends of expression regulation in 'Golden Delicious' (dark grey) and Manchurian crabapple (light gray) at 0, 3, 7, 14 DAF (*x* axis). The expression levels are indicated on the *y* axis and are relative to the value at 0 DAF in 'Golden Delicious'. All the data were normalized to actin and *EF1α*. Bars represent the standard deviation for three biological replicates. Significant differences between 'Golden Delicious' and Manchurian crabapple are indicated (*p<0.05; **p<0.01).
