# Supplementary material for: Isoenzymes of the Flavonoid and Phenylpropanoid Pathways Show Organ-Specific Regulation during Apple Fruit Development
Source: Int J Mol Sci. 2023 Sep 20;24(18):14353. doi: 10.3390/ijms241814353 (PMC10532258; doi:10.3390/ijms241814353)
Supplement: Supplementary file 1 [file ijms-24-14353-s001.zip › Figure_S2.pdf]

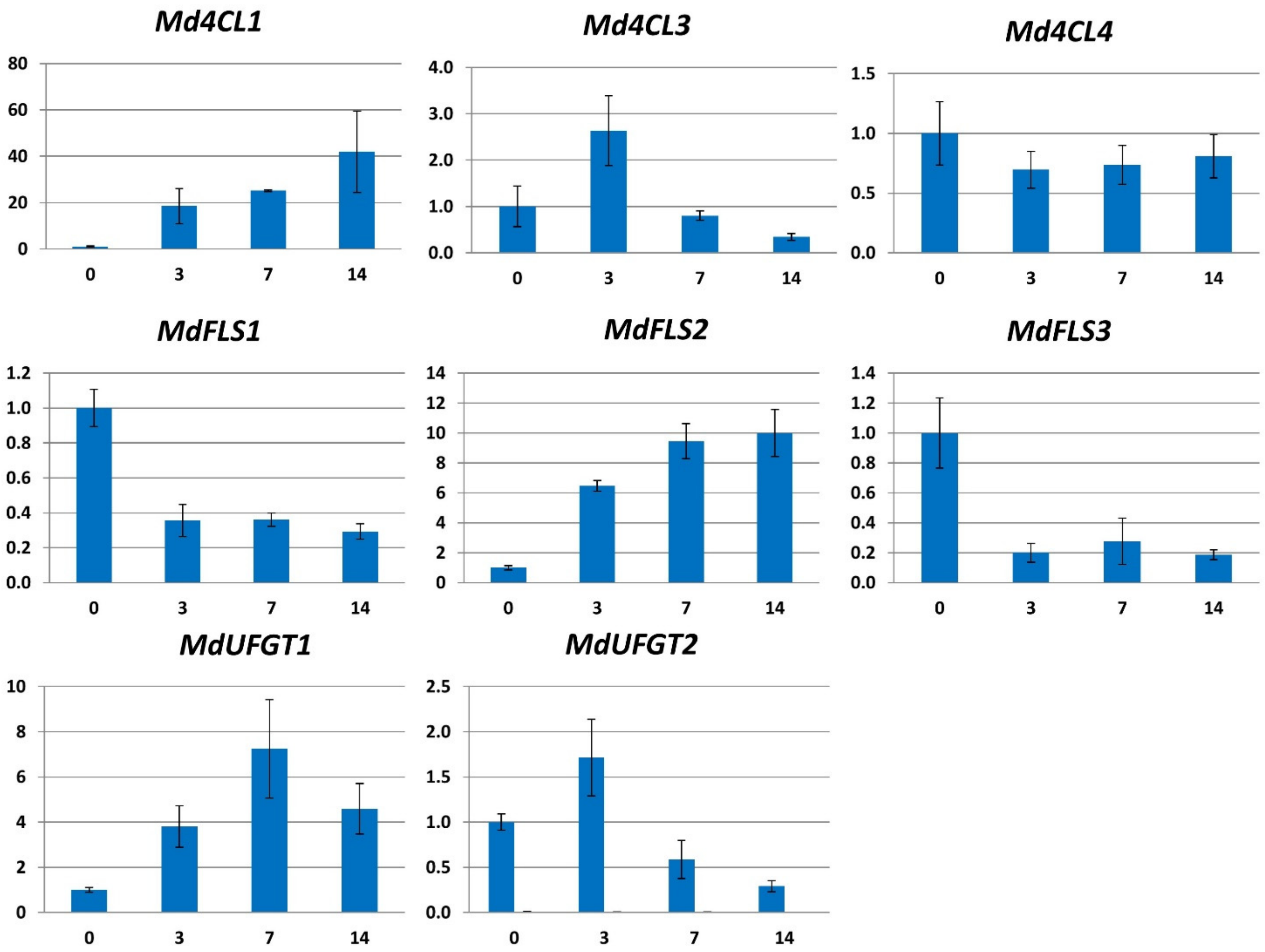

Figure S2. Relative expression levels of *Md4CL1, Md4CL3, Md4CL4, MdUFGT1, MdUFGT2, MdlAR1* and *MdLAR2* in 'Golden Delicious' at 0, 3, 7, 14 DAF. The relative expression levels are indicated on the *y* axis and are relative to the value at 0 DAF and normalized to actin and *EF1α*. Bars represent the standard deviation for three biological replicates.
