# Supplementary material for: Isoenzymes of the Flavonoid and Phenylpropanoid Pathways Show Organ-Specific Regulation during Apple Fruit Development
Source: Int J Mol Sci. 2023 Sep 20;24(18):14353. doi: 10.3390/ijms241814353 (PMC10532258; doi:10.3390/ijms241814353)
Supplement: Supplementary file 1 [file ijms-24-14353-s001.zip › Table S3_IJMS.pdf]

Table S3. List of sequences obtained from the qPCR cloned bands.

MdCHI1
GTTCCCGGGCAACTTTGCTTTGTTGCAGGGGAAACACCATGCTTTCCCACCATCGACTC
TAGAACTGCCTCGGAAAGTAGTTTGCTTTCTATCACTGCATTTGCAGCTTCCGGTATGG
ATGCATCTTTGGAGAAGCTAATCGTGAGTAATCCTTCAGGAGATTGTGTGAAGAGAAT
AGAGGGACCCGGTGGGAAGTTTTGATCTTTGAAAACATCAATGAACTGTTCAATGGCT
TTGCCTTCTAGATCAGTGTAAATTCCCACTGACTTCCAAAAGAATACACAATTCTCCGA
AACTTAAA

MdCHI2
AAAAACTTTCCCACCCGGCGCCTCCATTCTTTTCACGCAATCTCCCAAAGGATCACTAA
CGATTAGCTTCTCTAGAGATGCATCCGTACCTGAAGTTGCAAACACGGTGATAGAAAA
CAAACTACTTTCCGAGGCAGTTCTAGAGTCAATCGTTGGAAAGCACGGTGTTTCTCCTG
CAGCAAAGCAAAGTTTGGCCGCAAGGTTATCGGAATTGTTGAATGGGTGCAAGGAATC
TTATCGTGCTG

MdCHI3
CTCTGACAAGTTTCGGAGATTGCGTTGCCTTTTGGAAGTCAATCGGAATTTACACTGAT
GCAGAAGGCAAAGCCATTGAAAAGTTCCTTGAAGTCTTCAAAGATCAAAACTTCCCAC
CCGGCGCCTCCATTCTTTTCACGCAATCTCCCAAAGGATCACTAACGATCAGCTTCTCT
AGAGATGCATCCGTACCTAAAGCTGCAAACACGGTGATAGAAAACAAACTACTTTCCG
AGGCAGTTCTAGAATCGATCGTTGGAAAGCACGGTGTTTCTCCTGCAGCAAAGCAAAG
TTTGGCCACAAGGTTATCTAAATTGTTGAGAGA

MdPAL1
GGGGAAAGTGCTTCGTGTGTTGGCGTCAACAGAACCATAGAAGCCAAACCAGACCCAA
CTGCTGTTCCATTGACCAAGGCAAGCCCTTCTTTTGGTTGCAACTCAAAGAACCCACCA
TCAATCCCAGCTAGCTTAAAGGCATCAGAAGCGGTTAAGGTTTCCCCATTAGGTCCGGT
GGACTTGGAGTTAGGCCTTCCAATCAAAAGCA

MdPAL3
GGGGGGCTATTAGATTCTTGAACGCTGGAGTGTTTGGAAGTGCTACAGAATCAGGCCA
CACACTGCCACACCAAGCAACAAGAGCAGCCATGTTGGTCCGAATCAACACACTCCTC
CAAGGCTACTCCGGCATAAGATTCGAAATCTTGGAAGCCATCACCAAGTTCCTGAACA
ACAACGTTACTCCATGCTTGCCCCTACGCGGTACTATTACCGCCTCA

MdPAL4
GGGGGTGCTGCAAGATCCAGCATTCAAGAATCTAATTAGCTCCCTCTGAAGAGCTCCA
CCTTGCTTGGTTCTCCTATGCGAGGTTGCACCAAAGCCAGTGGTCACACCATAGCTGTC
TGTGCCTTTGCCCATGCTATCCATAACCCAATCACTGCTGGCCTTTACCCCAGCCCTTTT
GTCCTCCGACAGCTCCACCTTGGCACCGTCATGGTTGGCAATCGCAGCCACAACGTGA
GGGGCAGGGGAGCTGTCCGAGGATAAAGGCGGGGTAAAGGCACCTGATTGGTTTATG
CATAGCTGGGCCAGGACCACGCTATGGCGTGCCCTCCTTTGCCGCCTCCCTAGACCCCC
CCCTGCCCCCCCCAAGCCACCACCCCCG

Md4CL1
GGCCACACGGGCATGCATTTGTTTCTTGGTCGATCACGCATAAACAAGTAACAGACAC
CATGACCATTGCTTCCAGTTCCGTCGAAACTCAAAAGCCGGCAGACATACCTACCAAT
CTCATGCCGTCTGAGATTAATTCTACCTCTCAACAAAATCTAACCCAATTGCAACCCGC

CGCCTGCACCAACAATATTATTGATTCCACCACCGCCACCTCCACCGCCACTGCCACCA
CTAACCATGGGAATTAATATCAAAACCCC

Md4CL4
AAATCCGCTCTCTTTCTCAATTCTTATTTTTCTGTATATTTCAATCTGTTTATTGGTCGAT
ATATCACACACAAAGAAGC
AACAGTCACCATGATCTCCATTGCTTCTAATTCCGTTGAAACCCAAAAGCCGGCAGAC
ACATCTACCAATCTCATGCCTC
CTCTGATTAATTCTACCTCTCAACAAAACCTAACCCAATTGCAACCCCCCGCCTGCACC
AACAATATTATTGATTCCACC
ACCGCCACCGCCACCAGTAACCATG

MdLAR1
AATGGCTGCCTAATCGCCGTGTATCGATAGTAGTCTGAACATGAATTTGATCAACAGTC
CTGATTCTGATAGATTGATCAAGCACAAGTGGGAGTGACAGCCCTGGATTCGACGACG
GCGTTTTTGGTGGAGACGTTCTCCTCATGCACTGGCTCTAG

MdLAR2
CCATTTTTTCTGTAATGAATTCCAGCTTCCTTGTCCAGCAATAAAAATAAAATAAAAAA
TCAAATTAGTATCTTCCTCGAAGAAACCCTAGAAACCCTCCCCCTCCATACCAATTTTA
TAACAAGCTCACCCCCGAACTGTTGGAACATTTCTTTTCCTAGTAATAAATCCAGAACA
TGA

MdDFR1
ACCCGCGGCGACGACGATCCACTGGAGCGACGTTGAATTTTGCCTCTCCGTCAAGATG
ACTGGTTGGATGTACTTCGTTTCCAAGACTCTAGCTGAGCAAGCTGCATGGAAGTATGC
CAAAGAAAACAACATTGATTTCATCACCATTATCCCAACTCTTGTGATTGGTCCATTTC
TCATGCCATCCATGCCACCAAGCCTCATCACTGGACTTTCACCAATCACACGAACTAAA
TCA

MdDFR2
ACATAATTGGGCATCATCAGCAGGGCCAGTATGTTCACTTGGACGACCTCTGCCTTTCT
CACATTTATCTTTACGAGCATCCGAAAGCCGAAGGCCGTTACATTTGTTCGTCACATGA
TGCTACAATTCACGAACTTGTAAAAATGCTCAGAGAAAAATACCCCGAATACAATATA
CCCACAAAGTTCAAGGGCATCGACGACAACTTAGAACCAGTTCATTTTTCTTCAAAGA
AGTTGAGGGAGATAGGGTTTGAGTTCAAGTACAGCTTGGAGGACATGTTTGTGGGGGC
TGTCGATGCGTGCCGGGCAAAGGGCTTGATTCCGATTCCGATTCCGGCAGAGAAAACT
GAGGCTGCTGAGGAGAGCAACCTCGTTGATGTCAAAGTTGGTTA

MdUFGT1
ATCCATGCGGGAGAGTCATTTTCGGAACTTGGACTCGGTAATTTCCGGCATGCTACTTC
AGATGGGACGGCTCCTCCCCCGTGCCACCGCAGTTTTCATGAACGGCTTCGAAGAATT
GGAACTCCCCATACCAAACGACCTAAAGTCCAAAGTCAACAAACTCCTCAACGTAGGA
CCTTCCAACGTAGCATCCCCGCTGCCACCGCTGCCGCCATCAGATGCTTGCTTGTCATG
GCTAGACAAGCAACAGGCTCCATCCTCCGTCGTGTACATAAGCTTCGGGACAGTGG

MdUFGT2
TAGGGTATCGGAAGAGCTGCTAGCATAGCGGAGGCCCTGGAAGCCACAGGGGCTCCCT
TCTTGTGGTCGCTCAAGGACAACTTCAAGACACCGTTGCTGAACGAGTTCTTGACAAA
AATATTGTCAAAGGTGAACGGGATGGTGGTGCCGTGGGCTCCTCAGCCGCATGTCCTG
GCCCACGCTTCGGTCGGAGCTTTCGTGTCACATTGCGGCTGGAACTCGCTGCTGGAGAC

TATAGCCGGAGGGGTGCCCATGATTTGTAGGCCATTTTTTGGAGACCACAGGCTTAATG
CAAGGATGGTGGAGGACGAGTTGGAGATAGGGGTAAATGTGGA

MdC4H1
CGTACCATTGACACATCCTCGACGCGCAGCAGAAGGGAGAAATCAACGAGGACAATG
TTCTCTACATCGTCGAGAACATCAATGTTGCTGCTATTGAAACCACATTGTGGTCGATC
GAGTGGGGAATTGCCGAGCTAGTGAACCATCCTGAAATCCAGAAGAAGTTGAGGGAG
GAACTTGACGCTGTCCTCGGTCGCGGAGTTCAGATCACAGAGCCTGATGTTCAGAAAC
TTCCCCAAACACTGTTCGGAGCCACTGTGGGTACCAGGTCCTCCGTTACCGGTGGCATC
TCCTACTGTTTTTTGTTTTTTGTAT

MdC4H2
CCTGAAATCTCTCTCGATGGGACAGCGCAACCTCGTCGTCGTCTCCTCGACGGAGCTCG
CCAAGGAGGTGCTCCACACCCAGGGAGTCGAATTTGGGTCGAGGACCAGAAACGTCGT
CGTTGATATTTTCACCGGTAAAGGCCAGGACATGGTGTTCACCGTCTACGGCGAGCACT
GGCGGAAAATGAGGCGGATCATGACCGTTCCTTTCTTCACCAACAAGA
