# Supplementary material for: Isoenzymes of the Flavonoid and Phenylpropanoid Pathways Show Organ-Specific Regulation during Apple Fruit Development
Source: Int J Mol Sci. 2023 Sep 20;24(18):14353. doi: 10.3390/ijms241814353 (PMC10532258; doi:10.3390/ijms241814353)
Supplement: Supplementary file 1 [file ijms-24-14353-s001.zip › Table S5_IJMS.pdf]

Table S5. List of the putative transcription factor-binding motifs selected for the creation of a motif-abundance profile (MAP). A brief description of each binding motif is included as found on NewPLACE site (https://www.dna.affrc.go.jp/PLACE/?action=newplace).

MYB-related motifs

MYBCORE
Keyword: MYB; myb; dehydration; water; stress; flavonoid biosynthesis; leaf; shoot.
Description: Binding site for all animal MYB and at least two plant MYB proteins ATMYB1 and ATMYB2, both isolated from Arabidopsis; ATMYB2 is involved in regulation of genes that are responsive to water stress in Arabidopsis; A petunia MYB protein (MYB.Ph3) is involved in regulation of flavonoid biosynthesis (Solano et al.EMBO J 14:1773 (1995)); See S000355.

MYBST1
Keyword: MYB; myb; Myb.
Description: Core motif of MybSt1 (a potato MYB homolog) binding site; MybSt1cDNA clone was isolated by using CaMV 35S promoter domain A as a probe. The Myb motif of the MybSt1protein is distinct from the plant Myb DNA binding domain described so far.

EECCRCAH1
Keyword: low-CO2.
Description: "EEC"; Consensus motif of the two enhancer elements, EE-1 andEE-2, both found in the promoter region of the Chlamydomonas Cah1 (encoding a periplasmic carbonic anhydrase); Binding site of Myb transcription factor LCR1 (see Yoshioka et al, 2004); N=A/G/C/T.

MYB2CONSENSUSAT
Keyword: MYB; rd22BP1; ABA; leaf; seed; stress.
Description: MYB recognition site found in the promoters of the dehydration-responsive gene rd22 and many other genes in Arabidopsis; Y=C/T; K=G/T; See S000177 (MYB2), S000175(MYBATRD22).

MYBCOREATCYCB1
Keyword: Cyc; M phase; Myb.
Description: "Myb core" in the 18 bp sequence which is able to activate reporter gene without leading to M-phase-specific expression, found in the promoter of Arabidopsis thaliana cyclin B1:1 gene; the 18 bp sequence share homology with a sequence found in the *N.sylvestris* cyclin B1 promoter.

MYB1AT
Keyword: MYB; rd22BP1; ABA; leaf; seed; stress.
Description: MYB recognition site found in the promoters of the dehydration-responsive gene rd22 and many other genes in Arabidopsis; W=A/T.

MYBPLANT
Keyword: Myb; MYB; Myb305; AmMYB308; AmMYB330; flower; PAL; CHS; DFR; Candi; Bz1; phenylpropanoid; lignin; leaf; shoot.
Description: Plant MYB binding site; Consensus sequence related to box P in promoters of phenylpropanoid biosynthetic genes such as PAL, CHS, CHI, DFR, CL, Bz1; Myb305; M=A/C; W=A/T; See S000355; TheAmMYB308 and AmMYB330 transcription factors from *Antirrhinum majus* regulate phenylpropanoid and lignin biosynthesis in transgenic tobacco.

MYB1LEPR

Keyword: Pti4; ERF; PR; MYB.
Description: Tomato Pti4(ERF) regulates defence-related gene expression viaGCC box and non-GCC box cis elements (Myb1(GTTAGTT), G box(CACGTG)).

WRKY-related motifs

WBOXHVISO1
Keyword: sugar; SURE; patatin; WRKY; isoamylase; SUSIBA2.
Description: SUSIBA2 bind to W-box element in barley iso1 (encodingisoamylase1) promoter.

WBOXATNPR1
Keyword: NPR1; WRKY; WRKY18; disease resistance; SA; W box.
Description: "W-box" found in promoter of Arabidopsis thaliana (A.t.) NPR1gene; Located between +70 and +79 in tandem; They were recognized specifically by salicylic acid (SA)-induced WRKY DNA binding proteins; See S000142 (SQ=TTGACC); See S000310 (SQ=TTTGACY); A cluster of WRKY binding sites act as negative regulatory elements for the inducible expression of AtWRKY18. See also S000142.

WBOXNTCHN48
Keyword: W-box; W box; WRKY; elicitor.
Description: "W box" identified in the region between -125 and -69 of a tobacco class I basic chitinase gene CHN48; NtWRKY1, NtWRKY2 andNtWRKY4 bound to W box; NtWRKYs possibly involved in elicitor-respsonsive transcription of defense genes in tobacco; Y=C/T; see also S000442 (TGACT) and S000447 (TGAC).

WRKY71OS
Keyword: WRKY; GA; MYB; W box; TGAC; PR proteins.
Description: "A core of TGAC-containing W-box" of, e.g., Amy32b promoter; Binding site of rice WRKY71, a transcriptional repressor of the gibberellin signaling pathway; Parsley WRKY proteins bind specifically to TGAC-containing W box elements within the Pathogenesis-Related Class10 (PR-10) genes; See S000390 (TTGAC), S000442 (TGACT).

Light-responsive motifs

INRNTPSADB
Keyword: initiater; light-responsive transcription; TATA-less promoter; psaDb; Inr element.
Description: "Inr (initiator)" elements found in the tobacco psaDb gene promoter without TATA boxes; Light-responsive transcription of psaDb depends on Inr, but not TATA box.

IBOXCORE
Keyword: I box; I-box; rbcS; light regulation; light; leaf; shoot.
Description: "I box"; "I-box"; Conserved sequence upstream of light-regulated genes; Conserved sequence upstream of light-regulated genes of both monocots and dicots; See IBOX (S000124).

PRECONSCRHSP70A
Keyword: HSP; chlorophyl; MgProto.
Description: Consensus sequence of PRE (plastid response element) in the promoters of HSP70A in Chlamydomonas; Involved in induction ofHSP70A gene by both MgProto and light; S=G/C; Y=C/T; R=A/G;H=T/C/A; D=A/T/G.

10PEHVPSBD
Keyword: psbD; chloroplast gene expression; circadian rhythms; light regulation.
Description: "-10 promoter element" found in the barley (H.v.) chloroplast psbD gene promoter; Involved in the expression of the plastid gene psbD which encodes a photosystem II reaction center chlorophyll-binding protein that is activated by blue, white or UV-A light.

GATABOX
Keyword: ASF-2; GATA box; Cab; chlorophyll a/b binding protein; leaf; shoot.
Description: "GATA box"; GATA motif in CaMV 35S promoter; binding with ASF-2; three GATA box repeats were found in the promoter of Petunia(P.h.) chlorophyll a/b binding protein, Cab22 gene. Required for high level, light regulated, and tissue specific expression; conserved in the promoter of all LHCII type I Cab genes.

GT1CONSENSUS
Keyword: GT-1; light; TATA; TFIIA; TBP; HR; SAR; TMV; leaf; shoot.
Description: Consensus GT-1 binding site in many light-regulated genes, e.g., RBCS from many species, PHYA from oat and rice, spinach RCA and PETA, and bean CHS15; R=A/G; W=A/T; For a compilation of related GT elements and factors. GT-1 can stabilize the TFIIA-TBP-DNA (TATA box) complex; The activation mechanism of GT-1 may be achieved through direct interaction between TFIIA and GT-1; Binding of GT-1-like factors to the PR-1apromoter influences the level of SA-inducible gene expression.

Abiotic stress-related motifs

ABRELATERD1
Keyword: ABRE; etiolation; erd;
Description: ABRE-like sequence (from -199 to -195) required foretiolation-induced expression of erd1 (early responsive to dehydration) in Arabidopsis.

CCAATBOX1
Keyword: HSE (Heat shock element); CCAAT box.
Description: Common sequence found in the 5'-non-coding regions of eukaryotic genes; "CCAAT box" found in the promoter of heat shock protein genes; Located immediately upstream from the most distal HSE of the promoter; "CCAAT box" act cooperatively with HSEs to increase the hs promoter activity.

MYCCONSENSUSAT
Keyword: MYC; rd22BP1; ABA; leaf; seed; stress; CBF3; cold; CBF/DREB1;ICE1; RRE.
Description: MYC recognition site found in the promoters of the dehydration-responsive gene rd22 and many other genes in Arabidopsis; Binding site of ATMYC2 (previously known asrd22BP1); see S000144 (E-box; CANNTG), S000174 (MYCATRD22);N=A/T/G/C; MYC recognition sequence in CBF3 promoter; Binding site of ICE1 (inducer of CBF expression 1) that regulates the transcription of CBF/DREB1 genes in the cold in Arabidopsis; ICE1. This sequence is also known as RRE (Rresponse element).

ACGTATERD1
Keyword: ACGT; etiolation; erd.
Description: ACGT sequence (from -155 to -152) required for etiolation-induced expression of erd1 (early responsive to dehydration) in Arabidopsis.

Biotic stress-related motifs

GT1GMSCAM4
Keyword: GT-1 box.
Description: "GT-1 motif" found in the promoter of soybean (Glycine max) CaMisoform, SCaM-4; Plays a role in pathogen- and salt-inducedSCaM-4 gene expression; See also S000198 (GT-1 consensus).

CACGTGMOTIF
Keyword: G box; G-box; rbcs; chs; ACGT element; adh; Bz-2; R-motif; STR;GT-1; GBF; elicitor; bZIP; napin; strictosidine synthase; cell;leaf; shoot; Pti4; ERF; PR.
Description: "CACGTG motif"; "G-box"; Binding site of Arabidopsis GBF4; C.roseus G-box binding factor 1 (CrGBF1) and 1 (CrGBF2) can act as transcriptional repressors of the Str promoter via direct interaction with the G-box; See S000345; Essential for expression f beta-phaseolin gene during embryogenesis in bean, tobacco, Arabidopsis; Tomato Pti4 (ERF) regulates defense-related gene expression via GCC box and non-GCC box cis-element (Myb1(GTTAGTT) and G-box (CACGTG)); A prominent hit by in silico analysis in both induced and repressed phyA-responsive promoters; Review by Terzaghi WB, Cashmore AR.

ASF1MOTIFCAMV
Keyword: TGACG; root; leaf; CaMV; 35S; promoter; auxin; salicylic acid; light; as-1; TGA1a, TGA1b; CREB; ASF1; TGA6; shoot; xenobiotic stress; SAR; SA; Disease resistance.
Description: "ASF-1 binding site" in CaMV 35S promoter; ASF-1 binds to two TGACG motifs; See S000023 (AS1); Found in HBP-1 binding site of wheat histone H3 gene; TGACG motifs are found in many promoters and are involved in transcriptional activation of several genes by auxin and/or salicylic acid; May be relevant to light regulation; Binding site of tobacco TGA1a; TGA1a and b show homology to CREB; TGA6 is a new member of the TGA family; Abiotic and biotic stress differentially stimulate "as-1 element" activity.
